# Dynamics of serum immunoglobulin G and total protein concentrations in dairy calves during the first 2 weeks of life

**DOI:** 10.3168/jdsc.2022-0236

**Published:** 2022-10-08

**Authors:** Alexandra Correa, Noelia Silva-del-Río, Rubia Branco-Lopes, Fernanda Ferreira, Ainhoa Valldecabres

**Affiliations:** 1 Veterinary Medicine Teaching and Research Center, University of California-Davis, Tulare, CA 93274; 2 School of Veterinary Medicine, Department of Population Health and Reproduction, University of California, Davis 95616; 3 Teagasc, Animal and Grassland Research and Innovation Center, Moorepark, Fermoy, Co. Cork, Ireland P61 C996

## Abstract

The aim of the present study was to describe the dynamics of serum IgG (determined with radial immunodiffusion) and total protein (TP; determined with refractometry) concentrations during the first 16 d of life. Secondary objectives were to evaluate the transfer of passive immunity (TPI) classification at d 1 of life as a conditional factor for the aforementioned dynamics, and to describe over time changes on calves' TPI classification. At a commercial raising operation, 36 calves (19 Holstein, 17 Jersey) were sampled immediately after arrival (d 1) and at d 4, 8, 12 and 16 of life, for serum IgG and TP concentration, and hematocrit determination (HCT). Transfer of passive immunity was categorized based on serum IgG (IgG-Poor: IgG <18 g/L; IgG-Good: IgG 18 to <25 g/L; IgG-Excellent: IgG ≥25 g/L) and TP concentrations (TP-Poor: <5.8 g/dL; TP-Good: 5.8 to <6.2 g/dL; TP-Excellent: ≥6.2 g/dL). Multiple linear regression was used to evaluate serum IgG and TP changes over time, considering the effects of time after birth, breed, HCT, and TPI classification at d 1 of life. At d 1, median serum IgG and TP concentrations were 29.9 g/L and 6.3 g/dL, respectively (interquartile ranges: 21.3–42.3 g/L and 5.6–6.7 g/dL, respectively). Dynamics of serum IgG and TP concentrations were conditional to TPI at d 1 of life. Serum IgG concentration declined over time for IgG-Excellent and IgG-Good calves (18.1 and 4.6 g/L, respectively), but remained constant for IgG-Poor calves. Serum TP concentration declined over time in the 3 TPI groups but it was more marked for TP-Excellent (27%) and TP-Good (19%) than for TP-Poor (14%) calves. At d 1, 83.3% of the calves were classified as IgG-Excellent or IgG-Good, whereas 77.8, 55.6, 41.7, and 58.3% of calves were classified within these categories at d 4, 8, 12, and 16 of life, respectively. Similarly, at d 1, 66.7% of calves were classified as TP-Excellent or TP-Good, whereas 47.2, 36.1, 25.0, and 2.8% were classified within these categories at d 4, 8, 12, and 16 of life, respectively. In summary, our results indicate that serum IgG and TP concentrations decline over 16 d of life, and the decline is associated with TPI classification at d 1 of life. Further studies are needed to determine the biological implications of serum IgG and TP decline after d 1 of life, and to elucidate the factors determining the different dynamics. Our results suggest that current thresholds for TPI classification should be interpreted carefully when the age of calves is unknown or outside the age range used to define those thresholds (>24 h to 7 d).

## Highlights

• Relative to those on d 1 of life, serum IgG and total protein (TP) concentrations decreased over time during the first 16 d.
• Serum IgG concentration dynamics (2 wk of life) vary based on IgG at d 1 of life.
• Serum TP concentration dynamics (2 wk of life) vary based on TP at d 1 of life.

## Graphical Abstract

**Figure:**
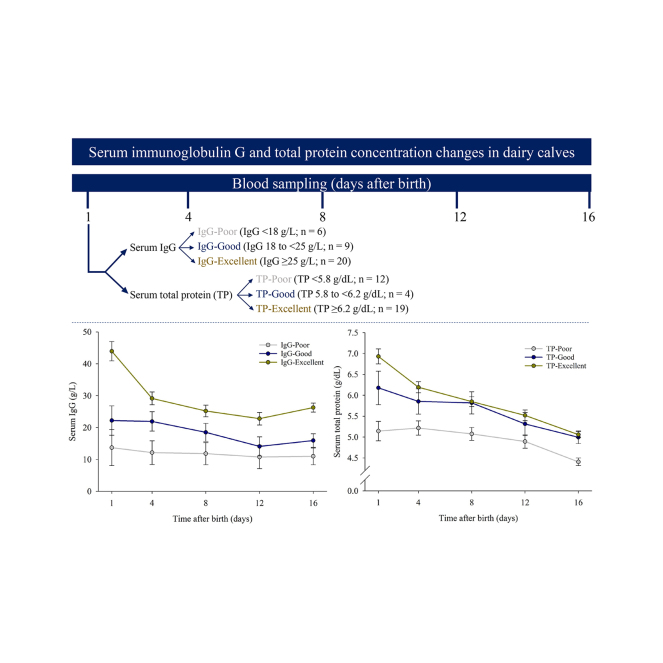
**Summary:** Transfer of passive immunity (TPI) at d 1 of life was categorized based on serum immunoglobulin G (IgG-Poor: IgG <18 g/L; IgG-Good: IgG 18 to <25 g/L; IgG-Excellent: IgG ≥25 g/L) and total protein concentrations (TP-Poor: <5.8 g/dL; TP-Good: 5.8 to <6.2 g/dL; TP-Excellent: ≥6.2 g/dL) for a total of 35 calves housed at a commercial raising operation. Overall, serum IgG (determined with radial immunodiffusion) and total protein (determined with refractometry) concentrations decreased during the first 16 d of life; however, the dynamics during these days varied based on TPI classification at d 1 of life.

Calves are born agammaglobulinemic (lacking maternal immunoglobulins) as the cow's cotyledonary synepitheliochorial placenta prevents transfer of passive immunity (Nocek et al., 1984; Peter, 2013). Furthermore, the calf immune system produces limited amounts of IgG during the first weeks of life (Husband et al., 1972; Sasaki et al., 1977; Devery et al., 1979). Thus, during early life, calves' immunity depends on the immunoglobulin absorbed from colostrum (Barrington and Parish, 2001), leaving colostrum as the only means for newborn calves to acquire passive immunity, as well as an important source of nutrients such as carbohydrates, fats, proteins, minerals, vitamins, and bioactive components (Morrill et al., 2012; Playford and Weiser, 2021; Valldecabres and Silva-del-Río, 2022).

Because failure of transfer of passive immunity from colostrum has been associated with deleterious effects on calf health (DeNise et al., 1989; Urie et al., 2018), its assessment is a widely recommended management tool to successfully rear healthy calves (Godden, 2008). For decades, industry guidelines defined failure of transfer of passive immunity as serum IgG concentration <10 g/L at 24 to 48 h of life [determined by radial immunodiffusion (**RID**); Godden, 2008]. A group of calf experts has recently revisited this recommendation and proposed 4 categories for transfer of passive immunity (**TPI**) classification and a wider time window for its determination. Serum IgG concentrations within 1 to 7 d of life of <10.0, 10.0 to 17.9, 18.0 to 24.9, and ≥25.0 g/L are considered to indicate poor, fair, good, and excellent TPI, respectively (Lombard et al., 2020). Although serum IgG concentration remains the reference method to determine TPI, this method is not practical on the farm. Alternatively, serum total proteins (**TP**) can be measured calf-side using refractometry as an indirect means to estimate serum IgG concentrations (Deelen et al., 2014; Elsohaby et al., 2015; Thornhill et al., 2015). The new guideline also provides equivalent serum TP concentration cut-off points: <5.1, 5.1 to 5.7, 5.8 to 6.1, and ≥6.1 g/dL to classify TPI as poor, fair, good, and excellent, respectively (Lombard et al., 2020).

The wider time window for TPI assessment proposed by Lombard et al. (2020; >24 h up to 7 d of life) had been previously suggested by others as a means to facilitate on-farm monitoring and increase the number of eligible animals in small to medium-size operations (McGuirk and Collins, 2004; Godden et al., 2019). However, based on our experience in large California calf raising operations, assessment of TPI is commonly performed at calf arrival (24 to 48 h of life). Few studies have described the dynamics of serum IgG and TP concentrations over the first days of life. Roadknight et al. (2021) reported that from d 2 to 8 of life, serum TP remained constant, but IgG increased from 19 to 24 g/L (n = 59). Wilm et al. (2018) reported a daily 0.7 g/L decrease in serum IgG concentration and stable serum TP concentration between d 1 and 10 of life (n = 12), and Pauletti et al. (2003) observed a decrease in serum IgG and TP concentrations from d 1 to 10 of life for calves with medium (20–30 g/L) or high (>30 g/L) levels of IgG at d 1 of life, and steady concentrations for calves with low initial levels of IgG (≤20 g/L; n = 20/group).

Given the scope of the TPI assessment recommendation and its common use in commercial settings and research trials, further understanding of serum IgG and TP dynamics is needed to optimize its applicability and interpretation. We hypothesized that serum IgG and TP concentration will decline during the first 16 d of life. Thus, the primary objective of this study was to describe the dynamics of serum IgG and TP concentrations during the first 16 d of life, and the secondary objectives were to evaluate whether TPI classification at d 1 of life was a conditional factor for the aforementioned dynamics and to describe over time changes on calves' TPI classification using direct (IgG determined with RID) and indirect (TP determined with refractometry) measurements.

All procedures carried out in this study were approved by the University of California Davis Institutional Animal Care and Use Committee (#21615). The study was conducted from December 2019 to February 2020 on a commercial operation raising 1,000 preweaning Holstein and Jersey female calves. All enrolled calves were born at the same source dairy, located <1 km away from the raising operation. Newborn calves were separated from their dams within 3 h after birth and housed in group pens bedded with almond and rice husks for 24 h before being transported to the calf raising operation. Within the first 24 h of life, calves were fed 6 L of pasteurized colostrum from multiparous cows (three 2-L feedings), and their navels were dipped with a 7% iodine solution. At the calf raising operation, calves were placed in individual plastic hutches wire-fenced to allow for 1.73 m^2^ of patio space (SSL Calf Hutch, AGRI-5001), bedded with almond and rice husks, and bottle-fed twice a day 1.9 L of milk replacer (26% CP:20% fat, American Calf Products) prepared to reach a 14% Brix reading. Water and starter (20% CP Starter; J.D. Heiskell & Co.) were bucket-fed ad libitum starting at 1 and 3 d of life, respectively. From 3 to 15 d of life, calves were bottle-fed 1.9 L of oral electrolyte solution in between milk feedings (55 g/L; Kool Water Calf Electrolyte; American Calf Products) as part of the calf raising operation management procedures.

A convenience sample of 36 female calves (19 Holstein and 17 Jersey) were sequentially enrolled in the experiment as they arrived at the raising operation (26 ± 5 h after birth; mean ± SD). Blood samples were collected at the same time (1100 h) on d 1 (arrival), 4, 8, 12, and 16 of life by jugular venipuncture into 2 vacuum tubes (10 mL; BD Vacutainer): 1 without anticoagulant for serum IgG and TP determinations and 1 containing K_2_-EDTA for hematocrit (**HCT**) determination. Samples were stored on ice and transported to the laboratory within 4 h after collection. Blood samples for serum IgG and TP determination were centrifuged at 1,430 × *g* for 20 min at 24°C. Indirect measurements of serum TP were performed using the TP scale on a handheld digital Brix refractometer (Misco DD-2). The remaining serum was transferred into 1.7-mL aliquots and frozen at −20°C for later IgG determination. Serum IgG concentration was determined using a single RID technique in accordance with manufacturer directions (Triple J Farms) within 4 mo after collection. Samples that exceeded the highest bovine reference serum IgG concentration were diluted with deionized water in a 1:1 proportion. Diameters of precipitin rings were measured using a digital caliper (calibrated at 0.01 mm) after 24 h of incubation at room temperature. The intra-assay CV was 4.8% for IgG RID determination. Samples for HCT determination were transferred into microhematocrit capillary tubes (Jorgensen Laboratories Inc.) and centrifuged at 2,000 × *g* at 24°C for 5 min at room temperature. The HCT value was measured using a reader microhematocrit card (LW Scientific).

All statistical analyses were performed in SAS (SAS Institute Inc.). Based on Lombard et al. (2020) using serum IgG concentration, TPI was categorized as follows: fair to poor (**IgG-Poor**: IgG <18 g/L), good (**IgG-Good**: IgG 18 to <25 g/L), or excellent (**IgG-Excellent**: IgG ≥25 g/L). Using serum TP concentration, TPI was categorized as follows: fair to poor (**TP-Poor**: <5.8 g/dL), good (**TP-Good**: 5.8 to <6.2 g/dL), or excellent (**TP-Excellent**: ≥6.2 g/dL). Changes in serum IgG and TP concentration over time were evaluated through multiple linear regression using the MIXED procedure. Variables considered as fixed effects in the multiple linear regression models were time after birth (1, 4, 8, 12, and 16 d), breed (Holstein and Jersey), HCT, and TPI classification at d 1 of life (poor, good, or excellent; based on serum IgG or TP concentration at d 1 of life). Repeated measures were modeled with time as the repeated measure, calf as the subject, and the variance-covariance structure selected based on the lowest Akaike's information criterion. Independent explanatory variables not statistically associated with the outcome (*P* > 0.10) were removed by stepwise backward selection. Pairwise interactions among breed, TPI classification at d 1 of life, and time were evaluated and removed at *P* > 0.10 when appropriate. Least squares means comparisons were conducted with the PLM procedure adjusted by Bonferroni or Dunnett (with d 1 of life as reference). Final model fit was assessed with residual plots. Statistical significance was declared at *P* ≤ 0.05. The accompanying figures were created with SigmaPlot (version 14.0; Systat Software Inc.). The interquartile range (**IQR**: 25th percentile–75th percentile) was used to describe the variables.

Median serum IgG concentration at d 1 of life was 29.87 g/L (IQR: 21.32–42.27 g/L). Serum IgG concentration was associated with time after birth (*P* < 0.001), TPI classification at d 1 of life (*P* < 0.001), and time by TPI classification at d 1 of life (*P* = 0.005). Compared with that at d 1 of life (26.63 ± 2.63 g/L), serum IgG concentration was lower at d 4 (21.08 ± 1.73 g/L; *P* = 0.03), d 8 (18.53 ± 1.61 g/L; *P* < 0.001), d 12 (15.90 ± 1.71 g/L; *P* < 0.001), and d 16 of life (17.75 ± 1.22 g/L; *P* = 0.002). Relative to that at d 1 of life, serum IgG concentration declined by 5.55 ± 2.04 g/L (20.8%) at d 4, 8.10 ± 1.83 g/L (30.4%) at d 8, 10.73 ± 1.92 g/L (40.3%) at d 12, and 8.88 ± 2.34 g/L (33.4%) at d 16 of life. Overall, serum IgG concentration within the first 16 d of life was higher for IgG-Excellent (29.49 ± 1.68 g/L; n = 21) than for IgG-Good (18.54 ± 2.57 g/L; *P* = 0.004; n = 9) or IgG-Poor calves (11.91 ± 3.15 g/L; *P* < 0.001; n = 6). No significant differences in serum IgG concentration were observed between IgG-Good and IgG-Poor calves (*P* = 0.34). Within the first 16 d of life, time effect on serum IgG concentration was conditional on TPI classification at d 1 of life (Figure 1A); relative to d 1 of life, serum IgG concentration declined 17.66 ± 2.68 g/L for IgG-Excellent (40.2%; *P* < 0.001) and 6.27 ± 4.10 g/L for IgG-Good calves (28.2%; *P* = 0.03), but the decline was not significant for those classified as IgG-Poor at d 1 of life (2.72 ± 5.02 g/L; 19.83%; *P* = 0.97). No associations between serum IgG concentration and breed or HCT were observed.

**Figure 1:**
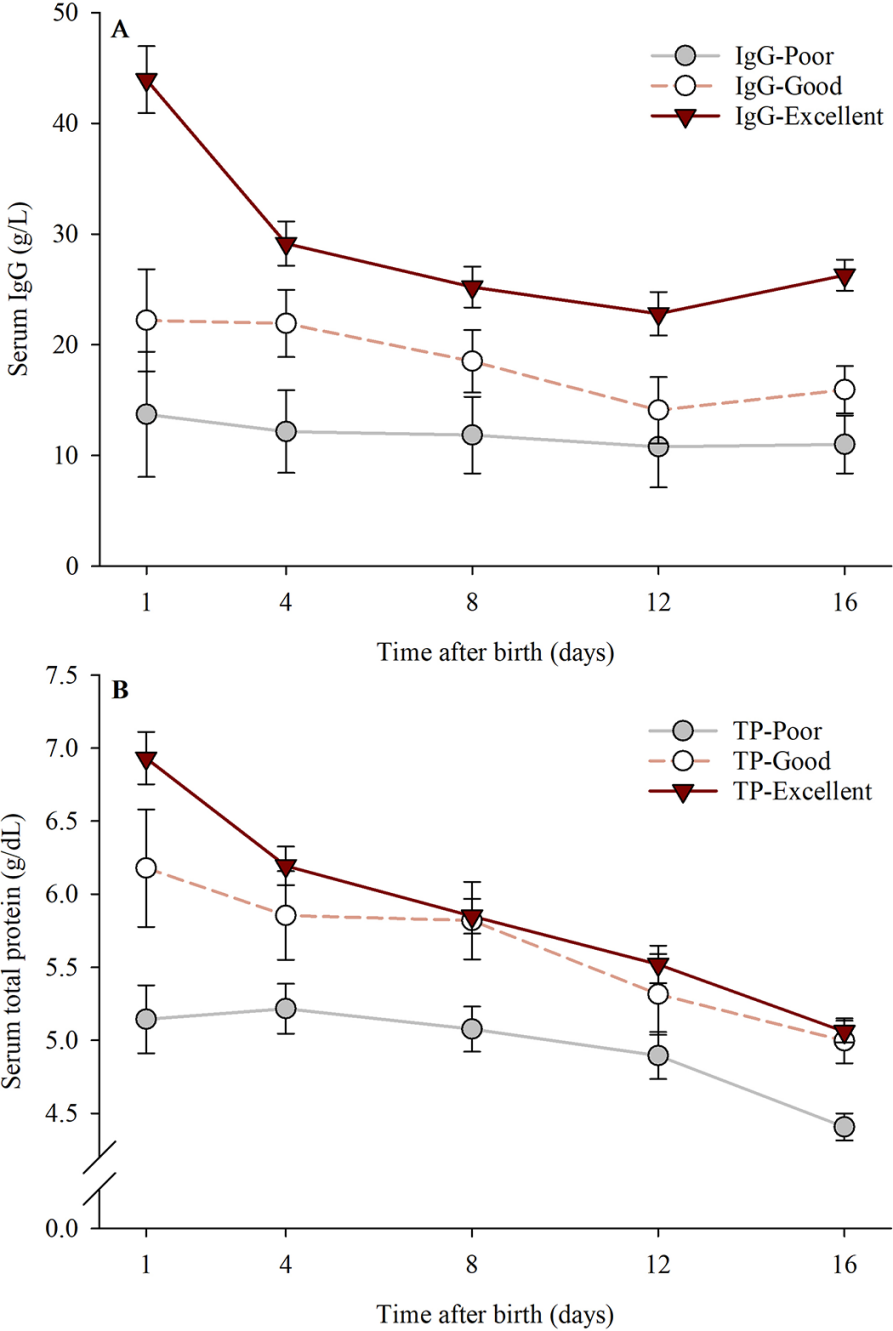
Serum IgG (A) and total protein concentrations (B) over the first 16 d after birth by passive immune transfer classification at d 1 for Holstein (n = 19) and Jersey (n = 17) calves. Calves were classified based on (A) serum IgG concentration at d 1 as follows: IgG-Poor (IgG <18 g/L; n = 6), IgG-Good (18 to <25 g/L; n = 9), and IgG-Excellent (≥25 g/L; n = 21); and (B) serum total protein concentration as follows: TP-Poor (total protein <5.8 g/dL; n = 12), TP-Good (5.8 to <6.2 g/dL; n = 4), and TP-Excellent (≥6.2 g/dL; n = 20). Fixed effects in the statistical models: time after birth (P < 0.001; panels A and B), passive immune transfer classification at d 1 (*P* < 0.001; A and B), hematocrit (HCT) (*P* = 0.11; B), HCT × HCT (P = 0.02; B), and time by passive immune transfer classification at d 1 (*P* = 0.005 and <0.001; A and B, respectively). Error bars indicate SEM.

Median serum TP concentration at d 1 of life was 6.3 g/dL (IQR: 5.6–6.7 g/dL). Similar to findings on serum IgG, serum TP concentration was associated with time (*P* < 0.001), TPI classification at d 1 of life (*P* < 0.001), and time by TPI classification at d 1 of life (*P* < 0.001) while adjusting by the HCT linear (*P* = 0.11) and quadratic (*P* = 0.02) effects. Compared with that at d 1 of life (6.08 ± 0.17 g/dL), serum TP concentration was lower at d 4 (5.75 ± 0.13 g/dL; *P* = 0.04), d 8 (5.58 ± 0.11 g/dL; *P* < 0.001), d 12 (5.24 ± 0.11 g/dL; *P* < 0.001), and d 16 of life (4.82 ± 0.07 g/dL; *P* < 0.001). Relative to that at d 1 of life, serum TP concentration declined by 0.33 ± 0.11 g/dL (5.4%) at d 4, 0.50 ± 0.10 g/dL (8.2%) at d 8, 0.84 ± 0.14 g/dL (13.8%) at d 12, and 1.26 ± 0.14 g/dL (20.7%) at d 16 of life. Overall, serum TP concentration within the first 16 d of life was 5.91 ± 0.11 g/dL for TP-Excellent (n = 20), 5.63 ± 0.24 g/dL for TP-Good (n = 4), and 4.95 ± 0.14 g/dL for TP-Poor (n = 12). Serum TP was significantly higher for TP-Excellent than for TP-Poor calves (*P* < 0.001); however, no significant differences on serum TP concentration were observed between TP-Good and TP-Poor (*P* = 0.06) or TP-Excellent calves (*P* = 0.92). Within the first 16 d of life, the time effect on serum TP concentration was conditional on TPI classification at d 1 of life (Figure 1B); relative to that at d 1 of life, serum TP concentration declined by 1.87 ± 0.15 g/dL for TP-Excellent (31.6%; *P* < 0.001), 1.18 ± 0.32 g/dL for TP-Good (21.0%; *P* = 0.002), and 0.74 ± 0.19 g/dL for TP-Poor calves (15.0%; *P* = 0.001), respectively. No statistical association between serum TP concentration and breed was observed.

Distribution of TPI classification over time based on serum IgG and TP concentrations is presented in Figure 2. Based on serum IgG concentration at d 1, 4, 8, 12, and 16 of life, the proportion of calves classified as IgG-Excellent was 58.3, 38.9, 30.6, 19.4, and 30.5%; IgG-Good was 25.0, 38.9, 25.0, 22.2, and 27.8%; and IgG-Poor was 16.7, 22.2, 44.4, 58.3, and 41.7% respectively. Based on serum TP concentration at d 1, 4, 8, 12, and 16 of life, the proportion of calves classified as TP-Excellent was 55.6, 25.0, 11.1, 11.1, and 2.8%; TP-Good was 11.1, 22.2, 25.0, 13.9, and 0.0% respectively; and TP-Poor was 33.3, 52.8, 63.9, 75.0, and 97.2%, respectively.

**Figure 2:**
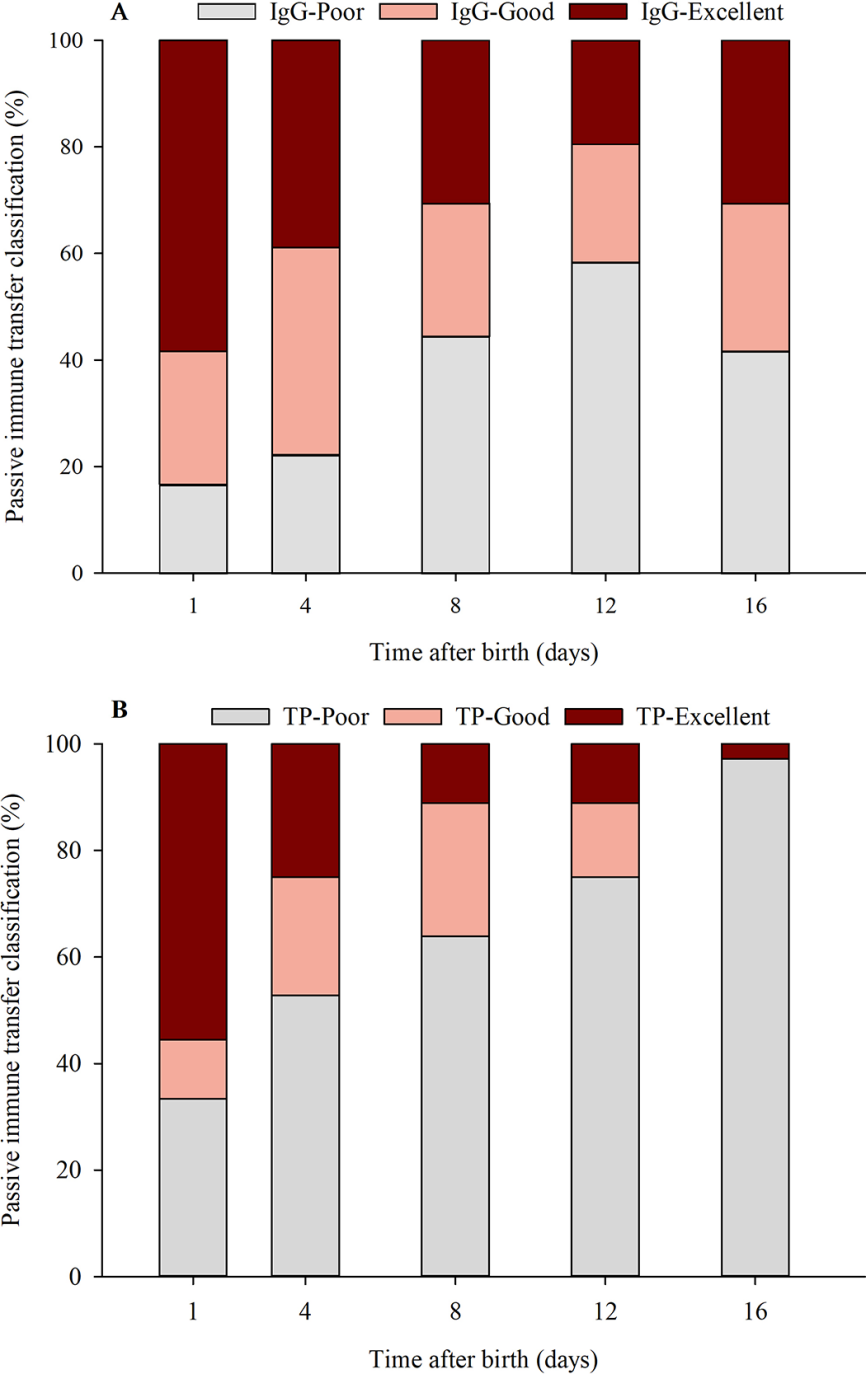
Passive immune transfer classification (%) within 16 d of life based on (A) serum IgG concentration (IgG-Poor: IgG <18 g/L; IgG-Good: 18 to <25 g/L; IgG-Excellent: ≥25 g/L) and (B) total protein (TP-Poor: total protein <5.8 g/dL; TP-Good: 5.8 to <6.2 g/dL; TP-Excellent: ≥6.2 g/dL).

On-farm monitoring of TPI is a highly recommended management practice to ensure the health and survival of calves (Godden, 2008). Based on the newly proposed TPI definition by Lombard et al. (2020), colostral acquired immunity can be evaluated in calves from 1 to 7 d of life using direct and indirect methods. We observed that serum IgG and TP concentrations decline over time, and the degree of this decrease is associated with the initially acquired immunity classification in 36 calves housed in a commercial farm.

The observed decline in serum IgG concentration over time is likely explained by IgG being metabolized or used in response to environmental immunological challenges and agrees with prior studies. Relative to d 1 of life, prior studies reported a decline in serum IgG concentration at d 7 to 8 of life [18.0% (IgG d 1 of life: 27.0 g/L; Lopez et al., 2020); 23.5% (IgG d 1 of life: 22.2 g/L; Wilm et al., 2018); 25.9% (IgG d 1 of life: 18.4 g/L; Husband et al., 1972)], except one study that reported a 20.0% increase in serum IgG from d 2 to 8 of life (Roadknight et al., 2021). Regarding serum TP changes over time, Wilm et al. (2018; n = 12) and Roadknight et al. (2021; n = 59) observed that serum TP concentrations remained constant from d 1 to 10 and from d 2 to 14 of life, respectively. The discrepancies in changes over time for IgG and TP in our study, as well as in prior studies, are difficult to explain. It is plausible that because IgG is just one component of serum TP (serum IgG represented 29, 38, and 64% of the TP at d 1 of life for IgG-Poor, IgG-Good, and IgG-Excellent calves), changes in TP over time are less obvious if other protein fractions remain relatively constant. Factors such as nutrition and dehydration may influence serum concentration of proteins; however, we accounted for this variation to the best of our abilities by determining and adjusting for the concurrent HCT. It should be noted that calves in our study were transported to the calf raising site (<1 km). However, some operations receive calves from distant sources, adding stress and increasing pathogen exposure during transportation (Minka and Ayo, 2010).

In our study, statistical differences in IgG and TP were reported with TPI classification over the 16-d study period except when comparing IgG-Good and IgG-Poor calves (+6.6 g/L), TP-Good and TP-Poor calves (+0.7 g/dL), and TP-Good and TP-Excellent calves (−0.3 g/dL). Consistent with our results, Pauletti et al. (2003) showed that TPI classification at d 1 of life was associated with the decline in serum IgG and TP concentration over time; for calves classified as high TPI (>30 g/L of IgG), serum IgG and TP concentration declined by 7.3 and 4.9% at 5 d of life and by 16.5 and 9.3% at 10 d of life, respectively; for calves classified as medium TPI (20 to 30 g/L of IgG), serum IgG and TP concentration declined by 6.4 and 1.7% at 5 d of life and by 14.4 and 3.9% at 10 d of life, respectively. Serum IgG and TP concentrations remained constant in low-TPI calves (<20 g/L of IgG). Endogenous IgG production is minimal in early life (Devery et al., 1979); thus, one possible explanation is that the lower initial availability of IgG translated into lower IgG use and catabolism or that, coincidentally, calves classified as IgG-Poor were less exposed to antigens. However, as accelerated endogenous IgG production has been documented in colostrum-deprived calves (Husband and Lascelles, 1975; Aldridge et al., 1998), IgG synthesis might have partially contributed to explain our findings. In our study, we limited sampling to 16 d of life, and we were not able to document time to IgG nadir with TPI classification. Additional factors such as breed, health, and management (e.g., hygiene, nutrition) may require further study to understand their implications on serum IgG and TP concentrations and dynamics.

In our study, the proportion of calves classified as TPI-Poor (<18 g/L) for IgG was 17% at d 1 and 44% at d 8; consistent with our results, Wilm et al. (2018) reported 36% at d 1 and 55% at d 8 (calculated from Figure 1). Thus, management decisions based on the proportion of calves within each TPI classification (e.g., payment for calves arriving at a feedlot, economic incentives to colostrum-feeding employees) should consider the age of calves at TPI evaluation. As current proposed thresholds were validated using calves from 24 h to 7 d of life (Urie et al., 2018; Lombard et al., 2020), future studies should examine whether the strength of the association between current thresholds for colostrum-acquired immunity classification (measured with IgG and TP) and calf performance (health and growth) is still adequate for calves aged 24 to 48 h.

Classifying calves based on TPI at d 1 of life led to categories represented by few calves, given our initial sample size, and we caution the reader about this limitation. Also, it should be noted that calf management may have influenced the results presented herein. Regardless, the observed serum IgG and TP dynamics conditional on TPI classification at d 1 of life have potential to be biologically relevant and warrant further study. Currently accepted TPI thresholds were validated with calves from 24 h to d 7 of life. Thus, validation of current thresholds for IgG and TP at different ages will be informative, especially considering that large calf raising operations are assessing TPI on a specific day [e.g., arrival (at 1 d of life)].

We conclude that serum IgG and TP concentrations decline within the first weeks of life, and this decline is associated with TPI classification at 1 d of life. Current TPI classifications should be interpreted carefully when calf age is unknown or is outside the age range used in their initial validation.
